# Is Extended Duration of Dual Antiplatelet Therapy After Carotid Stenting Beneficial?

**DOI:** 10.1097/MD.0000000000001355

**Published:** 2015-10-09

**Authors:** Kai-Ming Jhang, Jing-Yang Huang, Oswald Ndi Nfor, Zhi-Hong Jian, Yu-Chun Tung, Wen-Yuan Ku, Yung-Po Liaw

**Affiliations:** From the Department of Public Health and Institute of Public Health, Chung Shan Medical, University, Taichung City (K-MJ, J-YH, ONN, Z-HJ, W-YK, Y-PL); Department of Neurology, Changhua Christian Hospital, Changhua (K-MJ); Department of Pharmacy, Taichung Veterans General Hospital (Y-CT); and Department of Family and Community Medicine, Chung Shan Medical University Hospital, Taichung, Taiwan (Y-PL).

## Abstract

The optimal antithrombotic regimen after carotid artery stenting (CAS) remains uncertain. We aimed to elucidate if long-term duration of aspirin plus clopidogrel after CAS would provide clinically relevant benefit.

Patients receiving CAS were identified from the National Health Insurance Research Database, Taiwan. The discharge date following CAS was defined as index date. The study participants were divided into groups according to the prescribed duration of antiplatelet after the index date. They included the insufficient (<30 days), moderate (30–41 days), and considerable (≥42 days) groups. The risk of ischemic stroke, composite vascular outcome, and death were interested outcomes. To eliminate event-related prescription change, all outcomes that occurred within 42 days were excluded. Follow-up started 42 days after the index date and was censored when an event occurred or at 6 months.

A total of 4903 patients received CAS from 2004 to 2011. The total participants recruited for analysis (n = 2829) included the insufficient (n = 688), moderate (n = 372), and considerable groups (n = 1769). The event rates of ischemic stroke (3.92, 2.69, and 2.77%, *P* = 0.30), composite vascular stroke (5.52, 4.03, and 4.41%, *P* = 0.42), and death (3.05, 2.42, and 2.32%, *P* = 0.58) were similar for each group. Cox regression did not demonstrate significant associations between antiplatelet duration and the outcomes of interest.

Long-term use of aspirin plus clopidorel after CAS did not decrease the risk of ischemic stroke, composite vascular events, or death during 6 months of follow-up. More research on the appropriate duration of post-CAS dual antiplatelet is essential.

## INTRODUCTION

Carotid artery stenting (CAS) is a treatment of choice in patients with symptomatic or asymptomatic severe carotid artery stenosis.^1,2^ To prevent thromboembolism, dual-antiplatelet therapy is recommended before and after CAS. In patients receiving coronary artery intervention with bare metal stents, longer duration of dual antiplatelet (>1 month) has been proven to reduce the risk of adverse ischemic event.^3,4^ However, the optimal antithrombotic regimen for CAS remains uncertain.^2,5^ The society for vascular surgery and American stroke association guidelines suggested that dual-antiplatelet therapy with aspirin plus clopidogrel or ticlopidine, should be prescribed for at least 30 days after CAS.^1,6^ However, the 30-day duration came from the experience gained by experts in clinical trials of patients undergoing CAS and not conscientious studies.^7,8^ Therefore, the current use of antiplatelet after CAS is quite heterogeneous. Results from a study conducted in the United States reported 4 to 6 weeks or 2 months of dual antiplatelet after CAS in 30% of the centers; 3 months in 40% and 6 months in other centers.^9^

Until now, no study has discussed the suitable duration of dual antiplatelet therapy following CAS. We aimed to elucidate if long-term duration of aspirin and clopidogrel following CAS would provide an extra clinically relevant benefit.

## METHODS

### Data Source

This retrospective cohort study was conducted using data obtained from the National Health Insurance Research Database (NHIRD) and National Death Registry Database (NDRD), Taiwan. The National Health Insurance covers over 99% of the 23 million residents in Taiwan. The NHIRD contains comprehensive health care information, including diagnoses, prescriptions, and information on inpatient and outpatient care. The NDRD was linked to NHIRD through anonymous identification numbers to confirm date of death. This study was approved by the Institutional Review Board of the Chung-Shan Medical University Hospital, Taiwan.

### Patient Identification

Patients who received carotid stenting from 2004 to 2011were identified using the international classification of diseases, 9th revision, clinical modification (ICD-9-CM) 433.0–437.1 and the operation code 39.90. In general, 4903 patients were included in the study. The discharge date following CAS was defined as index date. The participants were then divided into 3 exposure groups according to prescribed duration of aspirin plus clopidogrel (Anatomical Therapeutic Chemical (ATC) code: B01AC04) after the index date. They included the insufficient group (<30 days), moderate group (30–41 days), and considerable group (≥42 days). Exclusion criteria included patients with stent complications (ICD-9-CM: 996.74), acute myocardial infarction (AMI, ICD-9-CM: 410–411), acute stroke (ICD-9-CM: 430.0–437.9), active gastrointestinal bleeding (ICD-9-CM: 531.0–531.3, 532.0–532.3, 533.0–533.3, 534.0–534.3, 578.0) (n = 270) and those who received anesthesia/blood transfusion (n = 28), or died (n = 71) within 42 days after index date. Further exclusion included patients who were admitted for more than 14 days during operation (n = 861), those who were never prescribed clopidogel or aspirin (n = 520), those who had other CAS within 90 days (n = 195), and those with incomplete information (n = 129). Finally, 2829 patients were eligible for analysis. The potential confounders included sex, residential area, urbanization, age at CAS treatment, admission days while receiving CAS, comorbidities (DM (ICD-9-CM: 250), Hyperlipidemia (ICD-9-CM: 272.0), and Cancer (ICD-9-CM: 140–208)).

### Definition of Outcomes

The primary outcome was ischemic stroke (ICD-9-CM: 434–437.1) whereas the secondary outcomes were composite ischemic events including ischemic stroke or AMI and death. We also evaluated the incidence of symptomatic hemorrhage (ICD-9-CM: 430–432, 531.00–531.31, 532.00–532.31, 533.00–533.31, 534.00–534.31, 578) in all the groups. Each patient was followed up (in days) from the 42nd day after the index date until the end of the event onset or when it was censored. The censor point was the date when the associated event occurred or 6 months since follow-up.

### Statistical Analysis

The SAS software was used in our study. ANOVA and post-hoc analysis was used to compare the mean difference among groups for continuous variable whereas *χ*^2^ test was used for nominal variables. The hazard ratio and 95% confidence interval of outcome was estimated by Cox proportional hazard model. *P* value < 0.05 was considered statistical significance.

## RESULTS

A total of 4903 patients received CAS from 2004 to 2011. The final analysis included 2829 eligible participants. Table 1 shows basic characteristics of the study subjects categorized into insufficient (aspirin plus clopidogrel usage <30 days, n = 688), moderate (30–41 days, n = 372), and considerable groups (≥42 days, n = 1769). The mean usage days of considerable groups were 68.9 (standard deviation 28.7). Table 2 shows the major outcomes at 3 and 6 months after CAS for each study group. No statistical differences were observed among outcomes in all the groups. However, longer duration of post-CAS dual antiplatelet treatment had trend to have lower risk of ischemic stroke, composite vascular events, and death. The risks of symptomatic hemorrhage were similar between groups. Table 3 shows cox proportional model to predict risk factors for ischemic stroke at 3 and 6 months since follow-up. Duration of antiplatelet treatment was not associated with ischemic stroke. The only significant predictor was “longer admission days (>7 days)” while receiving CAS (HR = 1.96, *P* = 0.024). Table 4 shows the estimated hazard ratios for all outcomes by different durations of antiplatelet treatment. In model 1, the moderate group was used as reference whereas in model 2, the insufficient group was used. Adjustments were made for age, sex, admission days, geographic area, urbanization, and comorbidities. No significant associations were found between antiplatelet duration and outcomes of interest.

**TABLE 1:**
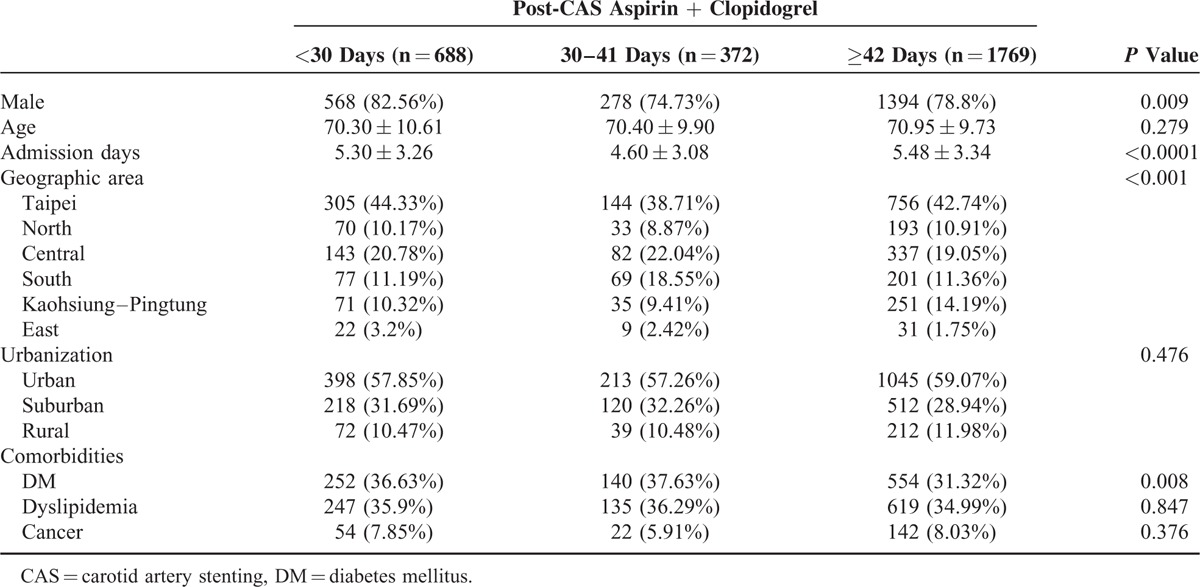
Basic Characteristics of Study Participants

**TABLE 2:**
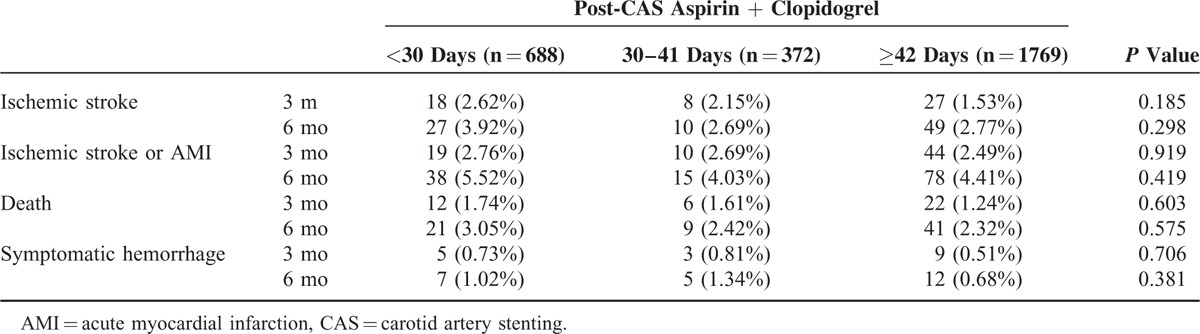
Major Outcome

**TABLE 3:**
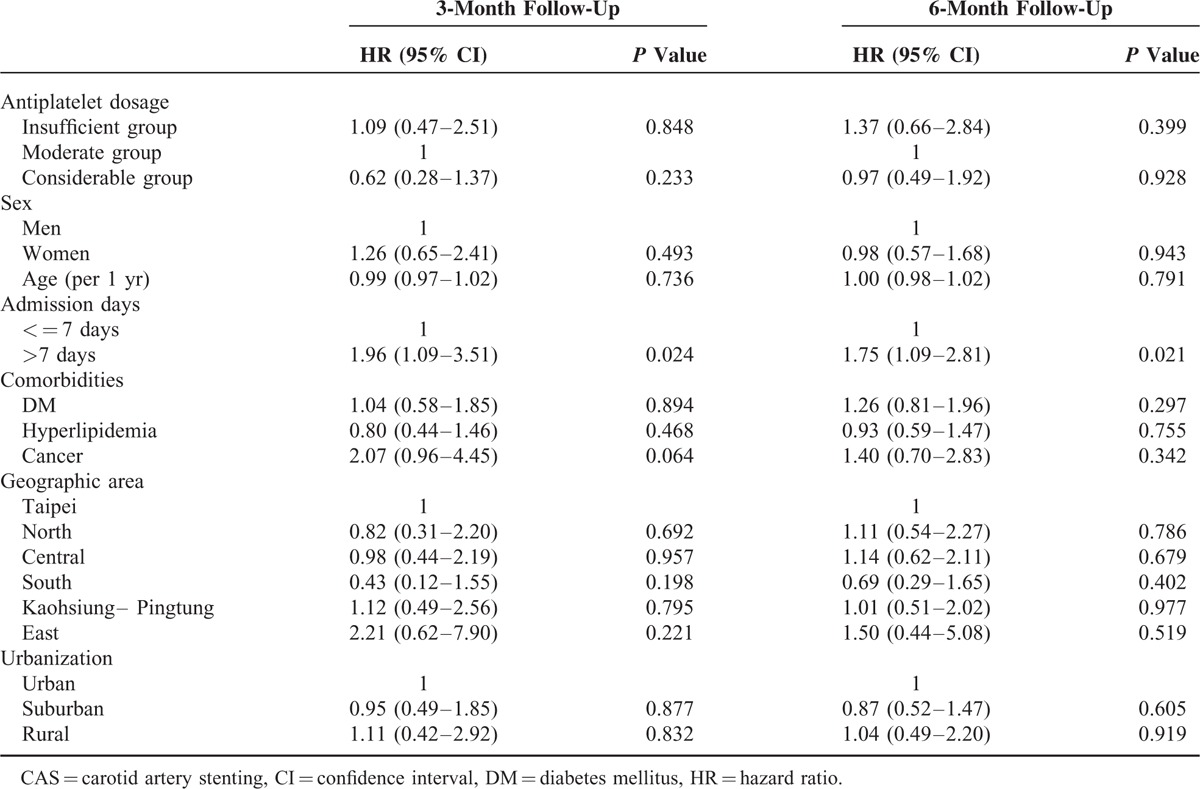
Cox Proportional Model to Estimate the HRs for Ischemic Stroke after CAS

**TABLE 4:**
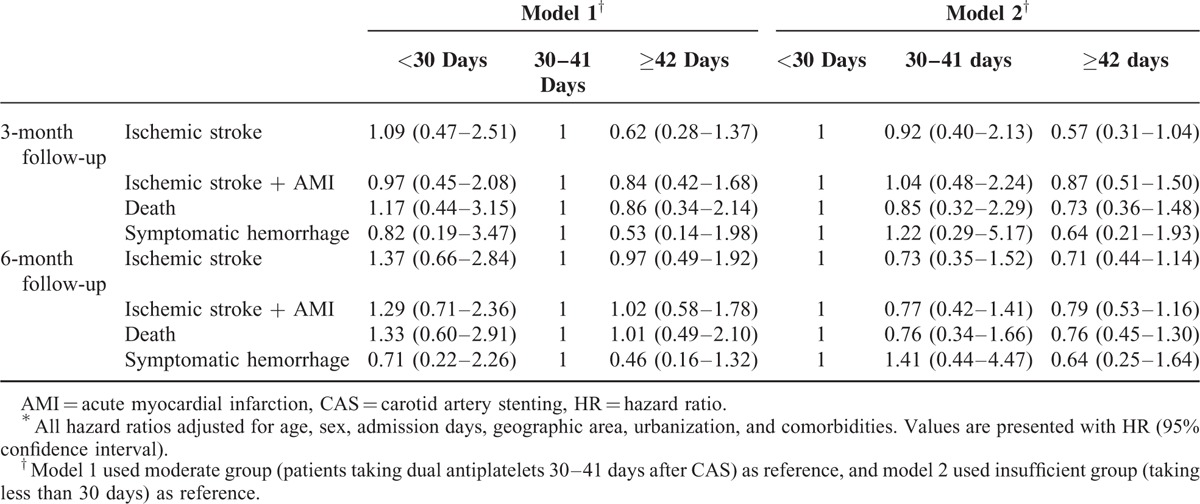
Estimated HRs for Major Outcomes by Duration of Antiplatelets After CAS^∗^

## DISCUSSION

To our knowledge, this is the first study to focus on the duration of dual antiplatelet treatment after CAS. The main finding of this study was that longer than 4 to 6 weeks of aspirin plus clopidogrel after CAS did not prevent ischemic stroke and vascular events. There are several possible explanations to this observation. First, most of the vascular events following CAS occurred within 30 days. Sakai et al^10^ found that the risk of any stroke was 5.7% within 30 days after CAS, whereas it was only 1.6% from 31 days to 1 year. Mo et al^11^ also published the rates of composite vascular event (stroke, AMI, and death) after CAS which were 1.5% within 30 days and 4.5% under mean 18.3 months of follow-up. Long-term outcomes after international carotid stenting study (ICSS trial) demonstrated that cumulative 1-year stroke rate was 9.5%; however, if it only included patients >30 days post-CAS, the rate was found to be 2.9%.^12^ Because most of the events occurred within 30 days, it is reasonable that extended antiplatelet therapy for more than 1 month did not provide obvious benefits. Second, the in-stent thrombosis rate is quite low after 30 days of CAS procedure. One of the main reasons for dual antiplatelet usage in stenting is to prevent in-stent thrombosis. Acute and subacute (within 30 days) in-stent thrombosis rate has been reported in 0.5–2% after CAS.^13,14^ However, only few reports have mentioned late stent thrombosis (>30 days).^15,16^ Most CAS patients received bare metal stent that has near complete neointimal coverage at 1 month,^17^ therefore sharply reducing the efficacy of dual antiplatelet treatment thereafter. Third, the efficacy of long-term dual antiplatelet in patients with high vascular risk is still questionable.^18^ A meta-analysis found that long-term (median 1.5–3.4 yr) combination of aspirin and clopidogrel in patients with stroke or transient ischemic attack did not prevent stroke and major vascular events but rather increased major bleeding.^19^ Most of the patients receiving carotid stenting were reported with cerebral ischemia. They probably did not benefit from extended duration of dual antiplatelet treatment based on current evidences.^19^

Longer hospitalization is the most significant risk factor for ischemic stroke following CAS. Longer admissions may lead to severe neurologic symptoms or complications that may result in higher rate of subsequent ischemic stroke. We did not observe high vascular events in individuals with insufficient antiplatelet treatment. This could possibly be attributed to the study design. Because this study was observational, we excluded all interesting outcomes (events) within 42 days after the discharge date to eliminate possible interference. That is, the duration of dual antiplatelet treatment was influenced only by the physician's choice of treatment. In this study, patients included in the insufficient group probably used dual antiplatelet for more than 30 days, considering that the index date was defined as the hospital discharge rather than the operation date. The NHIRD did not contain information on the operation date. The study design probably accounted for the high proportion (23.6%) of total participant in the insufficient group.

This study has several limitations. First, detection bias might have been possible through the use of ICD-9-CM. However, bias due to misclassification was ruled out considering that specific operation codes were used. The main outcomes of interest were severe diseases. Only the inpatient diagnostic codes were included in the analysis; hence detection bias was also minimized. Second, some potential confounders, including indication of CAS (symptomatic or asymptomatic), degree of carotid artery stenosis pre and post operation, and post-CAS neurological status, were not available. Nonetheless, possible factors such as admission days and comorbidities were adjusted. The third limitation has to do with the observational study design. The duration of antiplatelet treatment was not randomly assigned. In Taiwan, most centers have their perioperative protocol as in the USA.^9^ This study demonstrated that antiplatelet agents used in Taiwan vary.

In conclusion, the use of aspirin and clopidogrel lasting more than 4 to 6 weeks following CAS did not decrease the risk of ischemic stroke, composite vascular events, or death. The duration suggested based on current guidelines is probably suitable.^1,6^ More research is needed to provide more evidence on the appropriate duration of dual antiplatelet therapy after CAS.
